# Continuity and coordination of care: conceptual interface and nurses’ contributions

**DOI:** 10.1590/1980-220X-REEUSP-2022-0100en

**Published:** 2022-08-05

**Authors:** Mariana Timmers dos Santos, Bruna Marta Kleinert Halberstadt, Clediane Rita Portalupi da Trindade, Maria Alice Dias da Silva Lima, Gisele Knop Aued

**Affiliations:** 1 Universidade Federal do Rio Grande do Sul, Programa de Pós-Graduação em Enfermagem, Porto Alegre, RS, Brazil.; 2 Universidade Federal do Rio Grande do Sul, Escola de Enfermagem, Porto Alegre, RS, Brazil.

**Keywords:** Delivery of Health Care, Comprehensive Health Care, Continuity of Patient Care, Integration of Health Services, Nursing, Atención a la Salud, Atención Integral de Salud, Continuidad de la Atención al Paciente, Integración de los Servicios de Salud, Enfermería, Atenção à Saúde, Assistência Integral à Saúde, Continuidade da Assistência ao Paciente, Integração dos Serviços de Saúde, Enfermagem

## Abstract

This is a theoretical-reflective study, with the objective of discussing the concepts of continuity and coordination of care, its conceptual interface and nurses’ actions for its effectiveness in health services, based on international and national scientific publications. The concepts have been studied for decades and, although they are interrelated, they are used in a similar way, indicating a lack of conceptual understanding. The concept of continuity underwent paradigm shifts and began to adopt patients’ perspectives. Currently, it involves interpersonal, longitudinal, management and informational domains. Coordination consists of establishing connections between the possible elements involved in care. It is classified as horizontal and vertical and is organized into categories: sequential, parallel and indirect. Nurses stand out through actions aimed at coordination and continuity at different levels of care, which contributes to strengthening a cohesive and people-centered care. The interface between concepts indicates that, in order to achieve integrated and continuous services, continuity and coordination of care need to be interconnected and act together.

## RESUMO

Estudo teórico-reflexivo, com objetivo de discutir os conceitos de continuidade e coordenação do cuidado, sua interface conceitual e ações de enfermeiros para sua efetivação nos serviços de saúde, com base em publicações científicas internacionais e nacionais. Os conceitos são estudados há décadas e, embora sejam inter-relacionados, observa-se sua utilização de maneira semelhante, indicando falta de entendimento conceitual. O conceito de continuidade teve mudanças de paradigma e passou a adotar a perspectiva dos pacientes. Atualmente, envolve domínios interpessoal, longitudinal, gerencial e informacional. Coordenação consiste em estabelecer conexões entre os possíveis elementos envolvidos no cuidado. Classifica-se como horizontal e vertical e está organizada em categorias: sequencial, paralela e indireta. Enfermeiros destacam-se por meio de ações voltadas à coordenação e continuidade nos diferentes níveis de atenção, o que contribui para o fortalecimento do cuidado coeso e centrado nas pessoas. A interface entre conceitos indica que, para o alcance de serviços integrados e contínuos, continuidade e coordenação do cuidado precisam estar interligadas e atuar em conjunto.

## RESUMEN

Estudio teórico-reflexivo, con el objetivo de discutir los conceptos de continuidad y coordinación del cuidado, su interfaz conceptual y las acciones del enfermero para su eficacia en los servicios de salud, a partir de publicaciones científicas internacionales y nacionales. Los conceptos se han estudiado durante décadas y, aunque están interrelacionados, se utilizan de manera similar, lo que indica una falta de comprensión conceptual. El concepto de continuidad sufrió cambios de paradigma y pasó a adoptar la perspectiva de los pacientes. Actualmente, involucra dominios interpersonales, longitudinales, gerenciales e informacionales. La coordinación consiste en establecer conexiones entre los posibles elementos que intervienen en el cuidado. Se clasifica en horizontal y vertical y se organiza en categorías: secuencial, paralela e indirecta. Enfermeras se destacan por acciones dirigidas a la coordinación y continuidad en los diferentes niveles de atención, que contribuye al fortalecimiento de una atención cohesionada y centrada en las personas. La interfaz entre conceptos indica que, para lograr servicios integrados y continuos, la continuidad y la coordinación de la atención deben estar interconectadas y actuar juntas.

## INTRODUCTION

The concepts of continuity and coordination of care have been addressed in the literature in order to encompass current health challenges, which demand user continuous monitoring at different points of the Health Care Network (RAS – *Rede de Atenção à Saúde*). Continuity and coordination of care are linked to care quality and comprehensiveness^(1,2)^, which makes the conceptual understanding of these terms relevant and the analysis of how health professionals put them into practice.

The indiscriminate use of the terms continuity, coordination, integration and communication confuses their meanings both internationally^(3)^ and nationally^(2)^. It is believed that the close relationship between continuity and coordination of care contributes to difficulties in understanding and distinguishing between the terms, because continuity allows coordination of care, by establishing continuous interactions between the different professionals involved. On the other hand, coordination actions, such as the creation of protocols, patient flow and communication between services, provide an improvement in continuity of care^(4)^.

A Brazilian study that assessed how the interpretations on continuity of care have been configured in dissertations and theses in the health area, defended until 2019, identified that 50% were in the nursing area, 28.6% were in the collective health/public health and 21.4% were from other areas. It is also noteworthy that of the 186 selected studies, only 28 (15%) adopted continuity of care as an object of study, with the topic addressed mainly as an expected outcome in health practices. The definition of continuity of care was presented in only 53.6% of studies assessed. This can lead to the adoption of other terms in a similar way, influencing the interpretation of studies^(5)^.

In the international literature, there are different definitions of coordination of care. A comprehensive systematic review identified more than 40 definitions of this concept, which vary according to the different perspectives and actors involved. From the identification and combination of the central elements to the different perspectives, the conceptual model of McDonald et al. was established, widely adopted in national and international studies^(4,6–9)^. Still, the national literature presents divergence even regarding its denomination: coordination between levels of care or coordination of care^(2)^.

The concepts of continuity and coordination of care that have been adopted were established in studies carried out in developed and high-income countries^(10)^. An extensive literature review on the concepts of continuity and coordination and their relationships did not include studies from low- and middle-income countries and Latin Americans, due to the exclusion of studies that were not in English^(4)^. Therefore, there is still an important gap in studies that deal with continuity and coordination of care in these countries.

It is known that, in low- and middle-income countries, the realities are different, because there is greater fragility of health systems and limited resources. Continuity of care, then, ends up depending on informal care and family support. Moreover, they face additional challenges related to the health needs of people with chronic noncommunicable diseases (NCDs) in situations of greater vulnerability and itinerant populations, such as refugees, homeless people, among others^(10)^. Therefore, appropriation of concepts such as continuity and coordination of care in health care in these countries permeates different contexts and obstacles, evidenced by social, economic, demographic, epidemiological and cultural inequalities, which differ in developed and underdeveloped countries^(4)^.

In Brazil, the mismatch between the increase in chronic health conditions and work and management processes focusing on acute or chronic-acute conditions also demonstrate the fragmentation of care. To qualify care management and ensure continuity of care in different services, the Unified Health System (SUS – *Sistema Único de Saúde*) is organized in RAS, horizontally between different points of care, with different technological densities, with Primary Health Care (PHC) being the care coordinator^(5)^. This organization in networks, however, still encounters difficulties in several aspects related to operationalization, (dis)articulation of network points and adoption of a care model that aims at comprehensive care. The Brazilian National Policy of Primary Care (*Política Nacional de Atenção Básica*) brings continuity and coordination of care between the principles and guidelines for this operationalization in networks, however it does not explain the definition of these concepts^(6)^.

In this sense, this reflective study proposes to discuss the concepts of continuity and coordination of care, its interface and the actions of nurses for their effectiveness in health services. It is expected to broaden the understanding of the concepts of continuity and coordination of care and, also, that nurses and other health professionals can explore them in care practice, in order to contribute to comprehensive and qualified care provision.

Initially, conceptual topics on the continuity and coordination of care in different dimensions of health services and their interface will be presented. From this, the role of nurses in continuity and coordination of health care will be analyzed to compose the debate and reflections on the theme.

### Conceptual Approaches to Continuity of Care

The concept of continuity of care has been studied for decades by different researchers in the health area, having also been modified by contextual factors, such as the growing number of group practices, expansion of health sciences and the rise of PHC^(11,12)^.

Initially, around 1950, its concept was related to a medical attitude of continuous and solidary responsibility for patients, i.e., to have a reference professional for their care. Starting in the 1970s, the focus shifted to the relationship between care histories and the provision of coordinated, uninterrupted care^(3)^. Subsequently, multidimensional models were introduced to define continuity of care^(11–13)^.

The model of Haggerty et al., one of the most adopted in studies on the subject^(3)^, explains continuity of care from three dimensions: informational, relational and management. Informational continuity connects care between the different professionals who assist the patient and between one episode of care and another, and it is important to consider patients’ clinical history, beliefs and values. Relational continuity is the therapeutic relationship built between professionals and patients, and management continuity corresponds to the ability to offer different care that are complementary to each other, in a timely manner and without duplication^(12)^.

Deeny et al. developed a study that reviewed previous multidimensional models, upgrading to four continuity domains: interpersonal, longitudinal, management and informational. Interpersonal continuity involves subjectivity in the care relationship between patients and health professionals. Longitudinal continuity refers to a history of interactions with the same professional in a series of events. Management continuity translates into coordination processes, effective collaboration between teams and health services of different levels to provide cohesive care. Finally, informational continuity deals with the availability of clinical and psychosocial information in all consultations^(4,13)^.

The concept of continuity of care is often used interchangeably with the terms integration of services and coordination of care. There are some aspects that distinguish continuity of care from attributes, such as integration of services and coordination of care. One of these aspects is individual patient care and the other is care over time, regardless of duration^(12)^.

Recently, the World Health Organization defined continuity of care as the degree to which a series of health events are experienced by people as coherent and interconnected over time, consistent with their health needs and preferences^(4)^. Currently, continuity of care is guided by the paradigm that considers patients’ perspective, different from the previous one, which prioritized the view of health professionals. Professionals and patients tend to prioritize different aspects of continuity of care. In general, professionals prioritize workload and information continuity. In contrast, patients prioritize access to health services and support received^(14)^.

In this context, the assessment of continuity of care was developed by different approaches in the literature. Initially, was related to the frequency of consultations with the same physician, which led to the use of indexes and measures based on data on the use of health services, such as the Continuity of Care Index (COC), the Usual Provider Care Index (UPC) and the Sequential Continuity of Care Index (SECON)^(15)^.

However, the use of these measures does not identify individuals’ experiences regarding the care received. To assess continuity of care from patients’ perspective, there are different instruments, and among them, the Nijmegen Continuity Questionnaire (NCQ) and the *Cuestionario Continuidad Asistencial Entre Niveles de Atención* (CCAENA) stand out^(16)^. However, they are aimed at the care provided by physicians or specialists, which refer to the need to build or adapt instruments that assess the comprehensive continuity of care within the scope of the interprofessional care provided by the RAS.

A theoretical-reflective study, which analyzed continuity of care from the reference of symbolic interactionism, highlighting the subjectivity of the concept as a possible factor for incipient use in care practice. However, it reasserted that it is fundamental professionals’ awareness about its meaning and the understanding that continuity of care is in each professional’s action. Continuity of care results from a set of practices that depend on effective communication, good relationship between professionals and users, interdisciplinary work, articulation between different levels of attention and appropriate coordination of care^(1)^.

Despite its relevance, the Brazilian literature points out that studies on continuity of care are still scarce. This may be related to the conceptual adoption of the term continuity, more used in the international literature, while in Brazilian studies, the term longitudinality is more used. In a conceptual review on longitudinality and continuity of care, it was highlighted that the terms are used in a similar way in the literature, although they have conceptual differences. Longitudinality is one of the essential attributes of PHC, understood as patient follow-up by a multidisciplinary team over time. Continuity of care is associated with the succession of events and mechanisms for integrating information in meeting a problem or health needs, regardless of the establishment of lasting relationships^(17)^.

Although patients’ individual experiences can be aggregated at the collective level – between professional practices, health services and organizations, continuity of care is based on individuals’ experiences, not being an attribute of providers or institutions. Continuity is how individuals experience integration of services and coordination of care^(11,12)^.

### Conceptual Aspects of Coordination of Care

Initial discussions on coordination of care took place at the International Conference on Primary Health Care, held in Alma-Ata in 1978, which stated that PHC is responsible for the organization of health systems, later considered network organizer and care coordinator^(18)^. In Brazil, with the institutionalization of SUS, the Family Health Strategy (FHS) was implemented in order to strengthen PHC within the scope of the RAS, with coordination of care seen as a central link in system integration and organization^(6)^.

In the international context, coordination of care was defined as an essential attribute of PHC, articulated with first contact care, longitudinality and comprehensiveness^(19)^. Coordinating involves the organization of joint activities between two or more people, including users/family members and health professionals/services. It consists of establishing connections between the possible elements involved in primary, specialized and tertiary care, in order to fill gaps along the care trajectory, to meet individuals’ needs and preferences with quality^(20)^.

There is a positive association between the levels of coordination of care and the levels of quality of care provided in health services, i.e., the greater the coordination, the better the quality of care, constituting a differential for extensive and efficient care. Coordination actions collaborate to reduce errors in diagnoses and treatment measures, reduce waiting lines and unnecessary hospitalizations in highly complex services and reduce costs to the health system^(4,7)^.

Coordination of care encompasses different aspects, both assistance and management, of health care, aiming to meet individuals’ needs through the comprehensive offer of care, prioritizing the quality and continuity of care in the different services that make up the SUS priority networks^(6,20)^. To this end, coordination uses mechanisms and instruments for care planning, such as information exchange, flow definitions, referral and counter-referral systems, and patient monitoring by different professionals^(8)^.

Considering the different levels of health system integration, coordination of care can be classified as horizontal and vertical coordination. In this sense, horizontal coordination comprises health surveillance actions, programmed actions and spontaneous demand, interdisciplinary work and multidisciplinary team at the same level of care, while vertical coordination includes actions at different levels of health care^(21)^.

Also, coordination can be classified into three categories that provide interventions and, consequently, their qualification, namely: sequential coordination, also understood as transfer of care; parallel coordination, which is the planning of actions and the responsibility of different professionals in care; and indirect coordination, which encourages internal and external coordination through incentives, tools and/or continuing education for professionals^(4)^.

The establishment of coordination of care is supported by three pillars: informational coordination, which understands that all health information about individuals is available to professionals in all health services; clinical coordination, which implies a strengthened PHC, coordinating care at the various points of care; and organizational coordination, which concerns the network administrative flows and processes^(22)^.

Currently, there are instruments that make it possible to assess coordination of care in health services, such as the PCATool (Primary Care Assessment Tool), which measures the presence and extent of essential and derived PHC attributes, which has been adapted for use in Brazil^(23)^. It stands out as a low-cost assessment instrument that presents the conditions of health services. Research carried out with the PCATool assessed the attribute of coordination of care as satisfactory from professionals’ perspective, however, for users, the coordination score was unsatisfactory^(24)^.

In the Brazilian context, the Assessment Instrument for the Coordination of RAS by PHC (COPAS – *Instrumento de Avaliação da Coordenação das RAS pela APS*) is considered the only instrument that assesses the ability of PHC to coordinate RAS^(25)^. It is noteworthy that there are challenges related to the integration and coordination of health information about individuals, such as limitations in clinical and administrative management, integration of information systems, referral and counter-referral actions, considering that the country has different continental dimensions^(6)^. Such instruments contribute to the planning and implementation of new strategies to guarantee coordination of care in health services.

The lack of coordination actions affects continuity of care promotion^(26)^. Therefore, it is essential to know the strategies used by professionals to coordinate care and guarantee comprehensive, resolute and humanized care.

**Figure 1:**
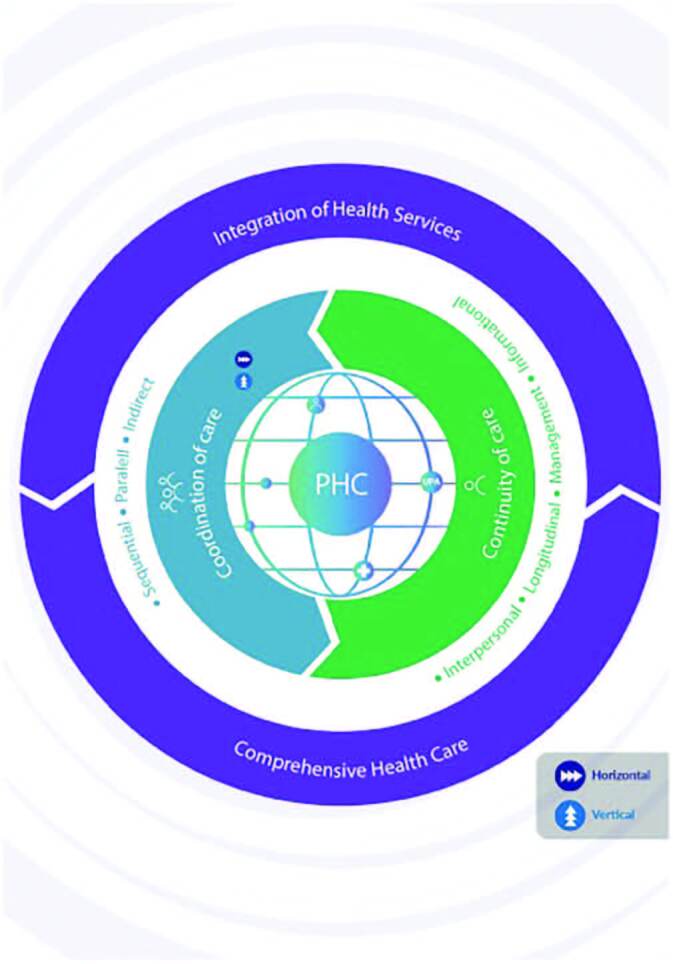
Interface between the concepts of continuity and coordination of care. Source: prepared by the authors, 2022.

From the conceptual explanations about continuity and coordination of care, it can be understood that there is an interface between these concepts. Through Figure 1, a visual representation of this interface was developed. The figure represents the RAS, its different points of care and the interface between the concepts of continuity and coordination of care, having PHC as a communication center, as it is the organizer of the network and performs coordination of care. In continuity of care, the icon representing a person refers to the perspective of individuals who receive care. In coordination of care, the icon with several individuals represents the perspective of the different professionals and services involved in care. To promote integration between the RAS services and comprehensive care provision, continuity and coordination of care operate interdependently in synergy. This simultaneous movement is represented in the figure by the arrows that surround the network.

### Nurses’ Work in Continuity and Coordination of Care

Patients are susceptible to experience discontinuity of care, when they go through changes in their health status or when moving from one service to another. Several practices are performed by health professionals to enable safe care transitions between different levels of health care. Nurses play an important role to ensure coordination and continuity of care for patients, developing actions that involve care planning at discharge, health education, articulation between services and post-discharge follow-up^(9,26,27)^.

Nurses have skills and competences for care management, including patients with complex demands, clinical and social assessment, and knowledge of health systems and services available for care follow-up. Furthermore, they play an articulating role, through communication and exchange of information between professionals and services^(26,28)^.

A national study, conducted with nurses working in private, public and philanthropic hospitals, identified that the main activities performed by nurses in the transition of care from hospital discharge are related to clarifying doubts of patients and family members during discharge guidelines, contacting the reference health team for continuity of care, identification of needs and discussion with patient and family about the care plan after discharge^(29)^.

Coordination of care in the transition from hospital to home, performed by nurses, also includes the execution of activities, such as medication reconciliation, guidance/education to patients and/or caregivers, post-discharge care follow-up, articulation and communication between the hospital and other health services and community support^(27)^.

Patients, especially those with complex health needs, require adequate planning and preparation for discharge. To this end, nurses are responsible for coordinating the discharge, through the management of care that patients needs, through team, multidisciplinary and interprofessional work^(30)^.

In Canada and Spain, liaison nurses, respectively, coordinate the hospital discharge of patients with complex needs. Before discharge, these nurses identify patients’ needs and preferences, planning together with the health team. Commonly, this planning includes patient care, the requisition of medical-hospital equipment, appointment scheduling, among others. Liaison nurses transfer information to community nurses or to the regulatory service via telephone or an integrated system. These professionals act as facilitators in the intervention of the different professionals and services that make up the RAS, so that patients and families reach the expected therapeutic results, strengthening continuity of care^(26,28)^.

Experience with the work of liaison nurses was carried out in a national study, demonstrating positive results in the context of RAS. Liaison nurses’ work was able to direct access to the health unit, qualify the communication between the different points of the RAS, contributing to preparing PHC to receive patients and meet their needs, in addition to reducing demand and return to more complex services^(31)^.

Coordination of care reflects positively in patient preparation to return home and, consequently, in the post-discharge results. Nurses, as the discharge coordinator, play a strategic role with the team, to facilitate patients and family to be able to perform care at home with autonomy, safety and quality^(30)^. It is important that hospital institutions allocate a professional to carry out coordination actions, without which continuity of care does not happen in its entirety^(26)^.

In the context of PHC, nurses’ work is complex and involves management, care and educational functions that require systematized planning and actions to coordinate population care^(6)^. Strategies, such as case management, care management and the multidisciplinary team, definition of flows, elaboration of protocols, use of electronic medical records, nursing consultations, continuing health education actions, acting in user referral and counter-referral between the RAS services, are interventions that contribute to the effectiveness of coordination and continuity of care^(32)^.

It is noteworthy that the term case management has been adopted since the 1960s in countries such as Canada, Spain and the United States and, in most of these locations, case management has been the nurses’ function. In the health system of Andalusia, in Spain, community case management nurses (CGE) have the role of assisting people linked to a health center and who need home care. These nurses must to preserve and improve quality of life of people who are disabled or at risk of suffering disabilities, as well as their caregivers, favoring the improvement of home care provided by the PHC team and improving coordination between PHC and the different levels of health care, in order to ensure continuity of care^(33)^.

It is understood that nurses develop actions aimed at coordination and continuity of care at different levels of health care. These actions demonstrate their contribution to strengthening person-centered care and boosting connections between professionals and patients, multidisciplinary teams and health services. However, there is a need to overcome some difficulties, considering the activities already performed by nurses in care and the shortage of professionals in the institutions. For this, the importance of creating specific positions to develop actions related to coordination and continuity of care is highlighted^(26,31)^.

## FINAL CONSIDERATIONS

The interface between the concepts of continuity and coordination of care leads to the understanding of these global priorities to redirect care in health services, according to people’s needs. Understanding these concepts and their compliance with care practices is essential for all health systems and professionals at different levels and services in the care provided in all life cycles.

Continuity and coordination of care are highlighted from the need to strengthen the integration between RAS services and promote comprehensive and patient-centered care. The concept of continuity of care has undergone transformations and is currently considered a multidimensional concept, focused on patients’ perception of coordinated care and in line with their needs. The concept of coordination of care is deeply discussed in the context of PHC as an element of integration and facilitator of continuity of care.

Continuity of care results from good coordination of care, while continuity of care actions feed coordination of care. Thus, they are interdependent and need to act synergistically to achieve integrated services and continuous care.

Nurses’ practices stand out as a possibility to visualize coordination and continuity in a more concrete way. Nurses act as care coordinators because, in addition to being close to patients and families during care, they play a leading role in problem solving and care management, discharge planning, health education actions, care transition and post-discharge follow-up, through communication and articulation with professionals and services. Given the above, the contributions of nurses in continuity and coordination of care can serve as a reference for other professionals.

Thinking about ensuring continuity and coordination of care is to overcome obstacles that permeate health systems, especially with regard to the services that constitute PHC and communication between the different RAS services. The possibilities lie not only in the limitations, but in the potential of the relationships between subjects, of effective communication between the services and professionals involved, in the provision of interprofessional care focused on people’s needs.

## Financial support

 This work was carried out with the support of the Coordination for the Improvement of Higher Education Personnel – Brazil (CAPES – *Coordenação de Aperfeiçoamento de Pessoal de Nível Superior*) – Financing Code 001 and financial support from the Brazilian National Council for Scientific and Technological Development (CNPq – *Conselho Nacional de Desenvolvimento Científico e Tecnológico*), Process 433997/2018-4.
